# Tremor after solid organ transplantation: Results from the TransplantLines Biobank and Cohort Study

**DOI:** 10.1111/ene.16412

**Published:** 2024-10-23

**Authors:** A. Madelein M. van der Stouwe, Niels L. Riemersma, Tim J. Knobbe, Daan Kremer, Svea Nolte, Danieke B. Plasmeijer, Antonio. W. Gomes‐Neto, Stephan J. L. Bakker, Gea Drost, Jan Willem J. Elting, TransplantLines Investigators, C Annema, SJL Bakker, SP Berger, H Blokzijl, FAJA Bodewes, MT de Boer, K Damman, MH de Borst, A Diepstra, G Dijkstra, RM Douwes, CSE Doorenbos, MF Eisenga, ME Erasmus, CT Gan, AW Gomes Neto, AM Posthumus, E Hak, BG Hepkema, J Jonker, F Klont, TJ Knobbe, D Kremer, HGD Leuvenink, WS Lexmond, VE de Meijer, GJ Nieuwenhuis‐Moeke, HGM Niesters, LJ van Pelt, RA Pol, AV Ranchor, JSF Sanders, MJ Siebelink, RJHJA Slart, JC Swarte, DJ Touw, MC van den Heuvel, C van Leer‐Buter, M van Londen, Charlotte A te Velde‐Keyzer, EAM Verschuuren, MJ Vos, RK Weersma

**Affiliations:** ^1^ Expertise Center Movement Disorders Groningen, University Medical Center Groningen University of Groningen Groningen the Netherlands; ^2^ Department of Neurology, University Medical Center Groningen University of Groningen Groningen the Netherlands; ^3^ Division of Nephrology, Department of Internal Medicine, University Medical Center Groningen University of Groningen Groningen the Netherlands; ^4^ Department of Neurosurgery, University Medical Center Groningen University of Groningen Groningen the Netherlands

**Keywords:** enhanced physiological tremor, solid organ transplantation, tacrolimus, tremor

## Abstract

**Background and purpose:**

Tremor is a frequent complaint of solid organ transplant recipients. We report on the largest population investigated with clinical neurophysiological methods. Our aim is to objectively establish the tremor prevalence and syndrome in the largest population of solid organ transplant recipients.

**Methods:**

Tremor was measured in heart, kidney, liver, and lung recipients, using accelerometers during rest, postural, and weight‐loaded conditions. The 95th percentile of healthy kidney donors' tremor amplitude was used as the cutoff to determine the presence of tremor in transplant recipients. Tremor frequency, frequency variability, and effect of loading were used to investigate enhanced physiological tremor as the likely tremor syndrome. Impact on activities of daily life was assessed, and correlations with tacrolimus blood levels were investigated.

**Results:**

Tremor was present in 52% of 246 transplant recipients, typically in postural positions. Mean tremor frequency was 6.1 (±2.0) Hz; mean tremor variability was 2.6 (±1.8) Hz. A frequency decrease upon loading was found in 83% of patients with tremor. Sixty‐five percent of patients met formal clinical neurophysiological criteria for enhanced physiological tremor. Tremor‐related impairment was present in 55% and correlated with tremor amplitude (ρ = 0.23, *p* ≤ 0.001). In a binominal regression analysis, tacrolimus blood levels were independently associated with tremor prevalence (*p* = 0.009).

**Conclusions:**

More than half of solid organ transplant recipients experience a tremor that best fits the syndrome of enhanced physiological tremor. This is the first objective study on tremor that has established a better understanding of the neurophysiological mechanisms of tremor in a large population of solid organ transplant recipients.

## INTRODUCTION

Patients who have received a solid organ transplant may report tremor as a bothersome symptom [1, 2]. Clinically, tremor is defined as involuntary, rhythmic movement of a body part [3]. Any limb can be affected, as well as the head and the voice. Many tremor syndromes exist, with a range of clinical features, etiologies, and treatment options [4]. Tremor can have an impact on a patient's daily life, as has been studied most extensively in patients with essential tremor, with complaints ranging from impairment in their ability to handle cutlery, to interference with the use of their phone [5, 6]. Some patients may need to change professions as a consequence [5]. Moreover, as it is such a visible symptom, patients are prone to embarrassment and social disability [5, 6, 7]. Given these findings, it is unsurprising that several studies describe surveys in which patients self‐report tremor in their top 10 most distressing symptoms after kidney or heart transplantation [2, 8].

The impact of the symptoms, as indicated by patients, warrants further investigation of tremor in organ transplant recipients. Currently, neither the objective prevalence nor the tremor syndrome is completely clear. Although previous efforts have been made, past studies have either been relatively small and specific to certain organs [9, 10] or have lacked proper investigation of the tremor syndrome from a neurological perspective [1, 2, 11], without the application of clinical neurophysiological methods such as electromyography or accelerometry. Subjective self‐reported tremor prevalence ranges from 40% to 79% in different studies [1, 2, 8, 11, 12]. Two studies employing objective clinical rating scales to investigate prevalence found tremor in 69% of 126 kidney transplant recipients [9] and 60% of 35 liver transplant recipients [10]. The occurrence of tremor among transplant recipients could be attributable to the necessity of calcineurin inhibitor use to prevent graft rejection. Unfortunately, this group of drugs is known to have neurotoxic effects. Therefore, with regard to the tremor syndrome, an enhanced physiological tremor would seem most probable, because this is the most frequent phenotype found in tremor induced by medication [13]. A decrease of the tremor frequency after adding a weight to the affected limb, a feature of enhanced physiological tremor, which was described in a single small study on calcineurin inhibitor‐induced tremor that incorporated accelerometry, also fits this hypothesis [10]. However, a diagnosis of enhanced physiological tremor is best supported by a combination of clinical neurophysiological features [14], which has not been reported in solid organ transplant recipients yet.

In this study, we aimed to investigate the prevalence of tremor in solid organ transplant recipients and characterize the tremor syndrome. We hypothesized that the tremor syndrome best fits an enhanced physiological tremor. By using both clinical and neurophysiological measurements, in contrast to previous literature, and studying the largest population described in the literature to date, we were able to provide a reliable prevalence and diagnosis of the tremor syndrome. This comprehensive approach enables us to further confirm and investigate any potential association with tacrolimus use.

## METHODS

### Transplant recipients and healthy participants

In this cross‐sectional study, we examined solid organ transplant recipients and kidney donors (aged ≥18 years) at the University Medical Center Groningen (UMCG; the Netherlands) between May 2016 and January 2019 as part of the ongoing, prospective, TransplantLines Biobank and Cohort Study (NCT03272841), which started in June 2015 [15]. The kidney donors served as a group of healthy participants. A prior medical history of tremor or Parkinson's disease was a reason for exclusion, in both donors and recipients, resulting in the exclusion of two patients because of essential tremor (*n* = 1) and Parkinson's disease (*n* = 1). The study was approved by the local medical ethical committee (METc 2014/077), adheres to the UMCG Biobank Regulation, and is in accordance with the World Medical Association Declaration of Helsinki and the Declaration of Istanbul. Written informed consent was obtained from all participants.

### Experimental setup

Healthy participants were examined during screening, and transplant recipients were examined ≥3 months after transplantation. During these visits, tremor was measured using accelerometry, and participants completed a functional disability questionnaire; these procedures are explained in more detail below. All data were collected by well‐trained investigators.

Participants were assessed during a resting condition (forearms resting on armrests, relaxed wrists) and four postural conditions that may elicit tremor: (i) forearms resting on armrests with wrists extended; (ii) pronated outstretched arms and relaxed wrists; (iii) pronated outstretched arms and wrists extended to neutral position; and (iv) hands in front of chest with shoulders abducted at 90°, fingers pointing toward each other. Moreover, postural tasks 1 and 4 were repeated as loaded conditions using a 1‐kg weight. These loaded conditions are relevant because some tremor syndromes are susceptible to change in frequency when a weight is attached to the tremulous limb [14, 16, 17]. Each task was performed for 30 s.

Movement was measured using wired bilateral triaxial accelerometers (UMCG) [18, 19]. The accelerometers were placed on the dorsal side of the hands, on the third metacarpal bone. A Kalman filter within the accelerometers corrected for gravitational artifact. Data were stored on hard disks and in Utopia (Utopia Data Management System v1.13.6, Research Data Support, UMCG).

To assess tremor‐related disabilities, a Dutch translation of part C of the Fahn–Tolosa–Marìn Tremor Rating Scale (FTM‐TRS‐C) was used [20]. This questionnaire consists of eight items covering activities of daily life. To evaluate symptoms associated with polyneuropathy, a questionnaire comprising six items, such as pain, numbness, and loss of balance, was employed. Additionally, medication at the time of the visit and medical history were registered.

### Data analysis

An in‐house‐written software program (LabVIEW 2014, National Instruments, Austin, TX, USA) was used to collect and analyze the accelerometry data of both transplant recipients and healthy participants. After visualization, data quality inspection was performed for each condition. Manual cursors were placed to select the data and to remove movement artifacts and improper task performance. After artifact elimination, a minimum duration of 10 s was required for each task. Next, a three‐dimensional acceleration vector was constructed from the triaxial acceleration data, which was integrated twice to obtain amplitude data over time. Mean subtraction and a 3‐Hz high‐pass Butterworth filter were applied after each integration step to correct for drift introduced by the integration and to remove low‐frequency artifact.

Two tremor parameters were calculated for each condition for each transplant recipient and healthy participant separately: (i) tremor amplitude in millimeters and (ii) tremor frequency in hertz (after fast Fourier transformation). The tremor frequency was determined by taking the frequency bin with the highest power value in the range of 3–15 Hz.

In the next step, healthy participants' accelerometry data were used to calculate a threshold to define the presence of tremor in an objective manner. The 95th percentile of the tremor amplitude in healthy participants was used as the cutoff value to define tremor in transplant recipients.

We continued the analysis in transplant recipients with objectified tremor by calculating three tremor features per transplant recipient: (i) tremor frequency was established as the mean tremor frequency across the unloaded tasks (in hertz); (ii) tremor frequency variability was calculated as the difference across the unloaded tasks (maximal tremor frequency—minimal tremor frequency, in hertz); and (iii) effect of loading was established by calculating the tremor frequency shift (in hertz) between the same postures in loaded versus unloaded conditions: forearms on armrests (loaded and unloaded), pronated outstretched hands (loaded and unloaded), and outstretched pronated arm and hand (loaded and unloaded), as is standard practice in our clinical neurophysiology laboratory.

Moreover, three clinical neurophysiological parameters were used to investigate enhanced physiological tremor as the likely phenotype: (i) tremor frequency > 6 Hz, (ii) frequency variability across multiple postural tasks > 1.75 Hz, and (iii) a decrease in tremor frequency of >1 Hz upon loading of the affected arm. Combination of these features results in a known useful diagnostic scoring tool; presence of two of the three criteria establishes enhanced physiologic tremor with a sensitivity of 84% and a specificity of 94% [14].

### Statistical analysis

Statistical analyses were conducted in SPSS version 23.0 (IBM, Armonk, NY, USA). Normality tests and descriptive statistics were performed. Correlation between tremor amplitude and FTM‐TRS‐C score was assessed using the Spearman rank test. We tested the correlation between tremor amplitude and tacrolimus full blood level using the Spearman rank test, as well as the correlation between tremor amplitude and time since transplantation, age, and polyneuropathy score. A multivariate binominal logistic regression analysis including all transplant recipients was performed with the presence of tremor in a patient as the dependent variable, and tacrolimus full blood level, time since transplantation, and polyneuropathy score as independent variables (predictors). A *p*‐value < 0.05 was considered to indicate statistical significance.

## RESULTS

### Transplant recipients and healthy participants

A total of 246 transplant recipients and 77 healthy participants were included. Demographic characteristics are presented in Table 1. Among transplant recipients, six (2%) patients received a heart, 135 (55%) patients received a kidney, 79 (32%) received a liver, and 26 (11%) received a lung. The median time interval between transplantation and participation in the study was 5 years (range = 3 months–38 years). A total of 157 (64%) transplant recipients were treated with tacrolimus, and 24 (9.8%) were treated with cyclosporine. Other immune suppressant medication included sirolimus, everolimus, mycophenolic acid, azathioprine, and prednisolone. Other relevant, potentially tremor‐inducing medications apart from immune suppressants were taken by 85 (35%) patients. Most common were amlodipine (*n* = 50), valganciclovir (*n* = 21), beta2‐sympathomimetic inhalers (*n* = 13), and several psychoactive drugs (e.g., citalopram, amitriptyline, paroxetine, *n* = 10).

**TABLE 1:** Demographic characteristics of transplantation recipients and healthy participants.

Demographic	Transplantation recipients, *n* = 246	Healthy participants, *n* = 77
Age, years	54 ± 13	54 ± 19
Gender	Male 152/female 88	Male 36/female 41
Transplanted organ		
Heart	6	
Kidney	135	
Liver	79	
Lungs	26	
Time since transplantation, years	5.0 (1.0–12.5)	
Tacrolimus blood level, μg/L	6.0 (4.1–7.7)	
Polyneuropathy score	1.0 (0.0–3.0)	

*Note*: Age is depicted as mean ± standard deviation. Time since transplantation, tacrolimus blood levels, and polyneuropathy score are depicted as median (interquartile range Q1–Q3).

### Tremor prevalence and characteristics

Tremor was present in 129 (52%) of the transplant recipients during at least one unloaded condition. Tremor‐evoking conditions were typically postural; the posture in which patients exhibited tremor most frequently was with pronated outstretched arms and hands, evoking tremor in 33% of transplant recipients. Tremor was observed least frequently at rest; only 5% of patients had a rest tremor. Of these 13 patients, 11 also had a postural tremor with a higher amplitude than the rest tremor, leaving two patients with an isolated rest tremor.

The mean tremor frequency was 6.1 Hz (SD = 2.0) in patients with established tremor (*n* = 129, 52%; Table 2). The proportion of patients with a tremor frequency > 6 Hz was 47%. Further analyses of tremor variability characteristics were performed in a subgroup (*n* = 80, 32%) of the transplant recipients, in whom tremor was present during at least two unloaded tasks, because this allows for assessment of variability over multiple tasks. The mean tremor variability was 2.6 Hz (SD = 1.8). The proportion of patients with frequency variability of >1.75 Hz across all unloaded conditions was 63%. The median effect of loading was a frequency change of −2.0 Hz (interquartile range [IQR] = −4.1 to 0.1) for the position with outstretched hands and a frequency change of −0.5 Hz (IQR = −2.2 to 0.4) for the position with both arms and hands outstretched. The proportion of patients with a frequency decrease of >1 Hz in at least one of the two loaded versus unloaded conditions was 83%. When combining all three neurophysiological features indicative of enhanced physiological tremor (i.e., tremor frequency > 6 Hz, frequency variability > 1.75 Hz, frequency decrease upon loading > 1 Hz), 65% of the patients scored positive for ≥2/3 points (EPT+).

**TABLE 2:** Clinical neurophysiological analysis of tremor in transplant recipients.

Tremor feature	Value
Tremor frequency	6.1 ± 2.0 Hz
Frequency > 6 Hz	47%
Tremor variability	2.6 ± 1.8 Hz
Frequency variability > 1.75 Hz	63%
Frequency change upon loading > 1 Hz—set 1	−2.0 (−4.1 to 0.1) Hz
Frequency change upon loading > 1 Hz—set 2	−0.5 (−2.2 to 0.4) Hz
Frequency decrease > 1 Hz in ≥1/2 set(s)	83%
≥2 positives for EPT Frequency >6 Hz Frequency variability >1.75 Hz Frequency decrease upon loading >1 Hz	65%

*Note*: Statistics are depicted as mean ± standard deviation, median (interquartile range Q1–Q3), or proportion.

Abbreviation: EPT, enhanced physiological tremor.

In an effort to identify a different tremor phenotype in the 35% of patients who did not meet neurophysiological criteria for enhanced physiological tremor (EPT−), we did a separate analysis of this patient group. We found the same profile of postural tremor with little rest tremor in both EPT− and EPT+ groups. Tremor frequency was lower in EPT−, with a median of 4.7 Hz (IQR = 3.3 Hz, range = 3.3–9.7 Hz) versus 6.5 Hz in EPT+ (IQR = 2.4 Hz, range = 3.8–9.6 Hz, Mann–Whitney *U*‐test, *p* < 0.001). Tremor variability across the different conditions was lower in EPT−, with a median of 0.9 Hz (IQR = 1.4 Hz, range = 0.1–4.0 Hz) compared to 3.4 Hz in EPT+ (IQR = 2.6 Hz, range = 0.2–6.6 Hz, Mann–Whitney *U*‐test, *p* < 0.001), with only 18% of EPT− showing a frequency variability above the specified 1.75 Hz. Polyneuropathy score, which was available in 51 (64%) of these patients, was not significantly different between EPT− and EPT+, with a median of 1.5 (IQR = 0.0–4.0) and 1.0 (IQR = 0.0–3.0), respectively (Mann–Whitney *U*‐test, *p* = 0.5).

### Correlations between objective tremor and subjective complaints

Median tremor‐related daily life impairment score as measured with the FTM‐TRS‐C was 1 (IQR = 0–2, range = 0–19) for transplant recipients. The proportion of transplant recipients who reported tremor‐related impairments (FTM‐TRS > 0) was 55%. Mean tremor amplitude across all unloaded tasks was positively correlated with FTM‐TRS‐C (Spearman ρ = 0.23, *p* ≤ 0.001).

### Medication, tacrolimus blood levels, and time since transplantation

The proportion of potentially tremor‐inducing medication other than immune suppressants was higher in recipients with tremor (43 vs. 28%, Wald test for equality of proportions, *p* = 0.023). As for immunosuppressing medication, mean tremor amplitude across all unloaded conditions correlated positively with tacrolimus blood levels (Spearman ρ = 0.37, *p* ≤ 0.001) and negatively with the time interval since the transplantation (Spearman ρ = −0.27, *p* ≤ 0.001). Tacrolimus full blood levels were negatively correlated with the time interval since transplantation (Spearman ρ = −0.55, *p* ≤ 0.001). There was no correlation with age. In the multivariate binominal logistic regression, tacrolimus blood levels (*p* = 0.009), but not time since transplantation, predicted whether a patient had a tremor (model significance *p* = 0.021). Tacrolimus odds ratio was 1.2 (95% confidence interval = 1.05–1.39), indicating that with each unit increase of tacrolimus blood level, the chance of tremor increases by 1.2 (i.e., 2‐U increase leads to 1.2^2^ = 1.44 increased chance of tremor, 5‐U increase 1.2^5^ = 2.5, etc.).

## DISCUSSION

In this study, our aims were to establish the objective tremor prevalence and the tremor syndrome in patients after solid organ transplantation.

First, we established that at least half of the transplant recipients exhibited tremor after solid organ transplantation (52%). To our knowledge, this is the first large study where tremor prevalence was measured objectively with accelerometry. Previously, the prevalence reported in studies investigating subjective self‐reported tremor ranged from 40% to 79% [1, 11, 12]. Furthermore, two studies assessed tremor prevalence objectively. One study found a tremor prevalence of 69% in a population 126 kidney transplant recipients, but they did not perform clinical neurophysiological tests [9]. Another study described the clinical tremor syndrome and reported an objective tremor prevalence of 60%. However, this study is limited by a small population size of 35 liver transplant recipients [10]. Here, we measured tremor amplitude using accelerometry, and defined the presence of tremor as an amplitude higher than the 95th percentile of what we found in our healthy participants. We believe this method to be a major strength of this study, as it takes into account normal variants of physiological movement in healthy participants and uses this physiological variation to define pathological tremor in an objective way. This way, we were able to establish tremor prevalence objectively using accelerometry in a large population of transplant recipients for the first time.

In terms of subjective measures, half of the transplant recipients reported impairments in daily life due to tremor, which is in line with previous reports [21]. There was a weak correlation between mean tremor amplitude and impairment score (FTM‐TRS‐C). This is not surprising, as correlations between objective clinician‐based measures and subjective patient‐based measures of tremor are known to be limited [6]. Our finding once again stresses the importance of incorporating both objective and subjective methods when assessing tremor severity.

Second, a picture emerges regarding the tremor syndrome, with several aspects fitting the syndrome of enhanced physiological tremor. First of all, tremor after organ transplantation can be classified as a postural tremor, not a rest tremor. Admittedly, the mean tremor frequency of 6.1 Hz is lower than expected in enhanced physiological tremor [14]. However, we did find tremor frequency to be variable across different postural positions, which does not fit with a stable central oscillator but is compatible with the peripheral machinal reflex component of enhanced physiological tremor. A frequency decrease upon loading, which again indicates an influenceable peripheral reflex component [22], was found in 83% of patients who had tremor. Together, these characteristics fit the tremor syndrome of enhanced physiological tremor. Formally, the majority of patients (65%) satisfy a set of clinical neurophysiological criteria for enhanced physiological tremor [14]. Out of interest, we performed a subanalysis of the patients who did not meet these criteria. In summary, we found the same profile of postural rather than rest tremor, but with significantly lower tremor frequency (median = 4.6 Hz) and frequency variability (median = 0.9 Hz). Although we realize this is to some extent to be expected given the criteria that separate the EPT− and EPT+ groups, the low tremor frequency stands out. This could fit with neuropathic tremor, in which low frequencies between 3 and 6 Hz are described [23, 24], as a second tremor syndrome encountered after solid organ transplantation. We were unable to substantiate this hypothesis with a higher polyneuropathy score in these patients, however, as this subjective questionnaire is not validated in transplant recipients, and a previous study found no correlation between subjective and objective polyneuropathy data [25], neuropathic tremor remains a possibility to be explored in future clinical studies.

Considering reasons for the development of enhanced physiological tremor in transplantation recipients, medication seems most likely. Organ transplantation recipients are frequently treated with calcineurin inhibitors such as tacrolimus and cyclosporine, which can lead to tremor as a side effect [13]. Tacrolimus is favored over cyclosporine [26, 27], but has previously been shown to be associated with more tremor [9, 11]. Also in our population, most patients were treated with tacrolimus, higher tacrolimus blood levels were associated with tremor amplitude, and higher tacrolimus blood levels increased the recipients' chances of having a tremor. The negative correlation between tremor amplitude and time since transplantation, the negative correlation between time since transplantation and tacrolimus blood levels, and the finding that time since transplantation had no independent predictive association suggest that the tremor is influenced by medication levels that are tapered in the period after transplantation. As the median time interval since transplantation was 5 years in our population, we speculate that the tremor prevalence in the first year after transplantation is even higher than the 52% that we report in this study. However, the time course would be best investigated with repeated assessments. Calcineurin inhibitors can also give rise to (poly)neuropathy as a side effect, which could lead to the development of neuropathic tremor. Apart from immunosuppressants, we found that the use of other medication with tremor as potential side effect was higher in patients with tremor. Future longitudinal studies are warranted to find evidence for the causality between medication use and tremor. Other tremor‐predisposing or interacting factors may be the patient's underlying illness, comorbidity, and their metabolic state.

This study has both strengths and limitations. The sizeable cohort containing several types of organ transplantation recipients, increasing the generalizability, can be viewed as a strength. The same holds for the incorporation of a truly objective measure for tremor prevalence, based on the 95th percentile of assessments in healthy participants, and the addition of a subjective measure of tremor severity. The additional measurement of surface electromyography in tandem with accelerometry would have allowed for the verification of rhythmic muscle bursts to further establish pathological tremor; however, we believe our approach using the 95th percentile of tremor amplitude as measured with accelerometry in a cohort of healthy participants as the cutoff between normal and pathological tremor circumvents this issue. A weakness is the lack of assessment before transplantation, which means we can give no information about changes due to transplantation procedures, but solely report our findings after transplantation. Our study would have been improved by a measurement before transplantation. Moreover, future research with repeated measurements at follow‐up would provide information on the tremor disease course after transplantation. Additionally, these measurements both before and after transplantation should include a clinical neurological assessment, with measures for bradykinesia, rigidity, and most importantly polyneuropathy, because some patients seem to suffer from a neuropathic tremor, a finding that would be substantiated by clinical signs. A clinical neurological examination was not deemed feasible beforehand given the extensive assessments in which transplant recipients partake in the TransplantLines Biobank and Cohort Study but would have been of great value in hindsight. In the current study, we evaluated the presence of polyneuropathy by means of a subjective questionnaire in a subsection of patients, but clinical neurological examination ideally accompanied by clinical neurophysiological examination remains the gold standard for establishing polyneuropathy.

To conclude, in this study we established tremor to be present in half of solid organ transplant recipients; the tremor characteristics best fit the syndrome of enhanced physiological tremor, with 65% of tremor patients meeting clinical neurophysiological criteria, and neuropathic tremor could be a second tremor syndrome after transplantation.

## AUTHOR CONTRIBUTIONS

**A. Madelein M. van der Stouwe:** Conceptualization; writing – original draft; investigation; formal analysis. **Niels L. Riemersma:** Investigation; writing – review and editing. **Tim J. Knobbe:** Resources; writing – review and editing. **Daan Kremer:** Resources; investigation; writing – review and editing. **Svea Nolte:** Resources; investigation; writing – review and editing. **Danieke B. Plasmeijer:** Investigation; writing – review and editing. **Antonio. W. Gomes‐Neto:** Resources; investigation. **Stephan J. L. Bakker:** Conceptualization; writing – review and editing. **Gea Drost:** Conceptualization; resources. **Jan Willem J. Elting:** Conceptualization; writing – review and editing; formal analysis.

## CONFLICT OF INTEREST STATEMENT

A.M.M.v.d.S. is supported by a grant from the Junior Scientific Masterclass Groningen (Mandema Stipendium 2018). T.J.K. and A.W.G‐N. are partly funded by a grant from Chiesi Farmaceuti (PA‐SP/PRJ‐2020‐9136). D.K. is funded by a grant from Astellas (TransplantLines Biobank and Cohort Study). None of the other authors has any conflict of interest to disclose.

## ETHICS STATEMENT

This study was approved by the Medical Ethical Committee of University Medical Center Groningen, reference number METc 2014/077. Informed consent was obtained. We confirm that we have read the journal's position on issues involved in ethical publication and affirm that this work is consistent with those guidelines.

## Data Availability

The datasets presented in this article are not readily available, because public participant data sharing is not included in the TransplantLines informed consent forms. Data requests are only legally allowed after approval by the TransplantLines Scientific Committee upon reasonable request, in accordance with the medical ethical committee allowance and the UMCG Biobank regulations. Requests to access the datasets should be directed to the corresponding author.
