# Corrigendum to “Tremor After Solid Organ Transplantation: Results From the TransplantLines Biobank and Cohort Study”

**DOI:** 10.1111/ene.70105

**Published:** 2025-05-30

van der Stouwe AMM, Riemersma NL, Knobbe TJ, et al. Tremor after solid organ transplantation: Results from the TransplantLines Biobank and Cohort Study. Eur J Neurol. 2024; 31:e16412. doi:10.1111/ene.16412

The ‘TransplantLines Investigators’ are missing in the Acknowledgement section and added as the last co‐author. The complete list can be found below:

TransplantLines Investigators: C Annema, SJL Bakker, SP Berger, H Blokzijl, FAJA Bodewes, MT de Boer, K Damman, MH de Borst, A Diepstra, G Dijkstra, RM Douwes, CSE Doorenbos, MF Eisenga, ME Erasmus, CT Gan, AW Gomes Neto, AM Posthumus, E Hak, BG Hepkema, J Jonker, F Klont, TJ Knobbe, D Kremer, HGD Leuvenink, WS Lexmond, VE de Meijer, GJ Nieuwenhuis‐Moeke, HGM Niesters, LJ van Pelt, RA Pol, AV Ranchor, JSF Sanders, MJ Siebelink, RJHJA Slart, JC Swarte, DJ Touw, MC van den Heuvel, C van Leer‐Buter, M van Londen, Charlotte A te Velde‐Keyzer, EAM Verschuuren, MJ Vos, RK Weersma.

The authors apologize for this error.

